# Compliance with iron folic acid and associated factors among pregnant women through pill count in Hawassa city, South Ethiopia: a community based cross-sectional study

**DOI:** 10.1186/s12978-019-0679-8

**Published:** 2019-02-08

**Authors:** Zemenu Yohannes Kassa, Tegibelu Awraris, Alemneh Kabeta Daba, Zelalem Tenaw

**Affiliations:** College of Medicine and Health Sciences, Hawassa University, Hawassa, Ethiopia

**Keywords:** Iron folic acid Compliance, Pill count, Ethiopia

## Abstract

**Background:**

Iron deficiency anemia during pregnancy is a public health problem across the globe that adversely affects maternal and perinatal outcome. World Health Organization recommends that universal iron folic acid supplementation of pregnant women. Therefore, the aim of this study was to determine compliance and identify factors associated with Iron folic acid among pregnant women.

**Methods:**

Community based cross-sectional study was conducted from November 01–December 30, 2015 on pregnant women from Hawassa city. An Interviewer administered questionnaire was used to collect data from 422 selected study subjects using simple random sampling technique. Data were entered in to Epi-Data version 3.1 and exported to SPSS version 20 for analysis. Bivariate and multivariable analyses were employed to test presence of association between dependent and independent variables. *P* value < 0.05 was considered as statistically significant.

**Result:**

In this study prevalence of compliance with iron folic acid was reported 38.3% (95%CI: 33.1, 42.5). Women who know the importance of iron folic acid had 6 times higher odds of compliance with iron folic acid than counterpart (AOR = 6.1, 95% CI: 3.53, 10.24).Pregnant women who develop complication during the previous pregnancy had 0.34 times lower odds of compliance with counterpart (AOR = 0.34, 95% CI: 0.16–0.76), experiencing iron folic acid related side effects during the previous pregnancy had 8.5 time higher odds to decrease compliance with than those did not experience iron folic acid related side effects (AOR = 8.5, 95% CI: 4.65.-15.35).

**Conclusion:**

In this study demonstrated that compliance with iron folic acid among pregnant women through pill count is low. Women who know the importance of iron folic acid, women who develop complications during previous pregnancy, experiencing iron folic acid related side effects during the previous pregnancy were independent predictor of compliance with iron folic acid. Health care providers shall strongly counsel the importance and side effect of iron folic acid before prescribing. The responsible bodies avail suitable iron folic acid pill and less side effect brands. Health care providers and health extension workers shall be monitored iron folic acid by pill count during their home to home visits.

## Plain English summary

Compliance with iron folic acid is a key means to prevent and control of physiological anemia during pregnancy. Iron folic acid compliance is stated that the woman take four tablet atleast per week (≥4 times per week) or for > 90 days at third trimester of pregnancy.

In this study the participants were asked through close ended and pretested structured questionnaire face to face interviews whether or not they took iron folic acid ≥4 times per week through pills count. Of the total 422, 383(95.3%) were married. The findings showed that 38.3% pregnant compliance with iron folic acid through pills count.

The possible determinants were: had complications at previous pregnancy, experienced of side effects at previous pregnancy and being informed of IFA’s importance.

In conclusion: compliance with iron folic acid during pregnancy is low in Hawassa city. This findings recommend that there is a need to give attention the way of iron folic acid taking during pregnancy.

## Background

Anemia remains a public health problem across the globe. It affected more than 2 billion people globally, accounting for 60,534 deaths in 2010 for reproductive age women. Pregnant women are predominantly at high risk of iron deficiency anemia (IDA) due to increase nutrient requirement during pregnancy [1, 2].

Globally, almost 47.4% preschool children, 41.8% pregnant women and 30.2% non-pregnant women were anemic [1, 3]. The prevalence of clinical anemia, anemia, ID (iron deficiency) and IDA in Ethiopia were 11.3, 30.4, 49.7 and 17.0% respectively [4].

Iron deficiency anemia contributes adversely pregnancy outcomes. Iron deficiency anemia during pregnancy is associated with an increased risk factor for maternal low weight gain, preterm labour, placenta previa, premature rupture of membrane, cardiac arrest, and hemorrhage, lowered resistance to infection, poor cognitive development and reduced work capacity. Similarly iron deficiency anemia on fetal and neonatal are increasing risk factor of prematurity, low birth weight, fetal distress which contribute to perinatal morbidity and mortality [5, 6].

Folic acid deficiency at conception is associated with risk factor of neural tube defects and other adverse pregnancy outcomes such as preeclampsia, malformations like facial clefts, spontaneous abortion, fetal death, fetal growth restriction, and preterm delivery. IFA supplementation and food fortification are now feasible strategy recommended to prevent such adverse pregnancy outcomes [7, 8].

WHO’s recommendation of IFA supplementation to pregnant women consider the existing magnitude of anemia: women live in area with 40% anemia to take 60mg iron plus 400μg folic acid on daily basis, in area with 20% anemia intermittent oral iron 120mg + 2800μg folic acid once weekly [9, 10].

A Study suggest that compliance with IFA is the major way of prevention and control of anemia and neural tube defect [11].

According to WHO’s compliance with IFA cut off pregnant woman is expected to take ≥90 IFA tablets on daily basis [12]. Studies conducted in India, Nigeria, and Sudan revealed that overall compliance with IFA tablets among pregnant women were 64.7, 65.9 and 91.1% respectively [8, 13].

According to the 2016 Ethiopian Demographic and Health Survey (EDHS) report in southern part of the country 4.2% of pregnant women took IFA ≥90 days [14]. In Ethiopia one of the strategies being implemented to prevent and control anemia and neural tube defect is universal supplementation of IFA for pregnant women. Studies done at different parts of the country revealed that 20.4% in Amhara region, and 74.9% in Tigray region, and 37.1% in South region were pregnant women compliance with IFA [15–17].

Studies showed that side effects, forgetfulness and shortage of supplement were major reason of non-compliance with IFA [15, 16].

Hence, assessment of compliance and identifying of factors associated with IFA is an important step to improve IFA intake among pregnant women. Despite the last fifteen years maternal and child health problems significantly decreased by applying different programs and interventions, anemia remains a substantial health problem in resource limited countries. Conversely another study confirmed that side effects of IFA had very limited influence on compliance, while by decreasing side effects of IFA may not be a successful strategy for improving compliance with IFA [13].

In Ethiopia minister of health being implemented among the strategies to prevent and control iron deficiency anemia and neural tube defects are universal supplement of IFA for pregnant women, meanwhile anemia remains unacceptable high. In Ethiopia compliance with IFA through pill count is limited data. Therefore, the aim of this study was to determine compliance and identify factors associated with IFA among pregnant women.

## Methods

### Study design, and study setting

Community based cross sectional study was carried out from November 01–December 30, 2015 among pregnant women who took IFA in Hawassa city.

Hawassa is administrative city of southern nation’s nationalities, people regional state (SNNPRS) and located 273km south from Addis Ababa. According to the city health department estimation report in 2015/16, there were 351,567 resident in Hawassa. From the total population170, 510(48.5%) were females. Women who were in child bearing age group (15–49) were 69, 769, from this, 12, 167 were pregnant. The city has 8 sub-city and 32 kebeles (smallest administrative unit), which have 83 public and private health institutions. One public referral and teaching hospital, one public general hospital,4 private primary hospitals,9 public health centers,17 health posts and 51 private clinics.

In Ethiopia IFA supplement is fundamental component of ANC. In the public health institution IFA supplementation is free of charge which is uncoated and the brand name is fefol.

### Sampling

Sample size was determined using single population proportion formula by taking the proportion of compliance with IFA through pill count 50%, at 95% confidence interval (CI) and an acceptable difference of 5%. Considering 10% non-response rate, the total sample size was 422.

There are 32 kebeles in the study area. Ten kebeles were selected using simple random sampling technique and home to home census was performed prior to one week data collection period to identify study participants in the third trimester of pregnancy who were supplemented to IFA tablets at first booking ANC for 3 months in the selected kebeles. Through census 1003 identified women who were supplemented to IFA tablets more than 3 months.

After having the list of all women in the third trimester of pregnancy who were supplemented to IFA tablets more than 3 months, with their respective houses codes were assigned for each pregnant women, and a new sampling frame was constructed. Simple random sampling technique was used. By using these numerical codes, study subjects were drawn by Open Epi, computer software.

### Inclusion and exclusion criteria

All women in the third trimester of pregnancy who were supplemented to IFA tablets at first booking ANC for 3 months in the selected kebeles were included. Women in the third trimester of pregnancy who were critically ill during data collection period, psychiatric problem, and on treatment of anemia were excluded.

**Operational definition**, **Compliance** defined as the willingness and ability of pregnant women to uptake IFA supplementation on daily basis. The level of compliance was assessed the following ways: the women took atelast 51 from 90 pill or the women took ≥4 times per week on daily basis (atleast 51 from 90 pill) through pill count [5].

**Obstetric complication during pregnancy** are vaginal bleeding, loss of consciousness, preterm labour, premature rapture of membrane and prolonged labour [18].

**Side effect of iron folic acid** are heart burn, nausea, vomiting, color change of stool and urine(black), constipation and intense abdominal pain [19].

### Data collection tool and quality control

Data collection was carried out in the community home to home. It was collected by face to face interview technique by using pretested questionnaire and pills were counted by direct observation. Questionnaire was developed by reviewing different existing literatures. First, the questionnaire was designed at English and then translated to Amharic and back to English to check consistency. A questionnaire was consisted of sociodemographic,obstetric and compliance with IFA variables. Pretest was conducted among 21 women (5% of the total sample size) and (Cronbach’s Alpha was 0.84) for internal validity of the tool in Yirgalem town. Based on the feedback and findings of the pretest, necessary correction and amendment was done before starting of the actual data collection. One day training was given for data collectors and supervisors on the content of questionnaire and its administration. Data were collected by ten diploma midwives and supervised by two BSc midwives.

### Data processing and analysis

Data were checked, coded and entered in to Epi-Data 3.1 and exported to SPSS Version 20.0 software. Data cleaning was performed to check accuracy and consistencies of variables. Descriptive statistics were computed to describe the study participants. Binary logistic regression model was computed to test presence of association between dependent and independent variables. To increase the power all variables with *p* value of less than 0.25 for bivariate logistic regression analysis were candidate for multivariable logistic regression analysis, and finally, significance was considered at *p* < 0.05 with 95% CI.

## Results

### Socio demographic characteristics of pregnant women

A total of 422 women in the third trimester of pregnancy was included in this study. Data from twenty (4.7%) were excluded from analysis due to incomplete.

The mean age of pregnant women were 25.7 ± 4.2SD. About 383(95.3%) of pregnant women were married, 125(31.1%) had college/university education, 162(40.3%) were house wives and 75(18.7%) were under poverty line (Table 1).

**Table 1:** General characteristics of pregnant women in Hawassa city, South Ethiopia, 2015

Variable		Number (*N* = 402)	Percent (%)
Age	< 18	3	0.7
	18–25	184	45.8
	26–35	211	52.5
	> 35	4	1
Marital status	Windowed	4	1
	Single	15	3.7
	Married	383	95.3
Religion	Others@	4	1
	Catholic	11	2.7
	Muslim	37	9.2
	Orthodox	168	41.8
	Protestant	182	45.3
Educational status	No formal education	39	9.7
	1–4 grade	50	12.4
	5–8 grade	109	27.1
	9–12 grade	79	19.7
	College/university education	125	31.1
Occupation	NGO employee	6	1.5
	Merchant	61	15.2
	Daily labor	67	16.7
	Government employee	106	26.4
	House wife	162	40.3
Monthly income^a^	Don’t know	26	6.5
	< 1313Eth birr (< 59.7USD)	75	18.7
	1313.5-9900Eth birr (59.7–450 USD)	301	74.9
Husband education	No formal education	3	0.7
	1–4 grade	21	5.2
	5–8 grade	57	14.2
	9–12 grade	101	25.1
	College/University education	220	54.7
Family size	> 6	21	5.2
	4–6	217	54
	< 4	164	40.8
Frequency of on TV medical advice follow-up	Once a week	72	17.9
	Once every two weeks	236	58.7
	Never	94	23.4
Frequency of reading medical magazine	Once a week	88	21.9
	Once every two weeks	91	22.6
	Never	233	55.5

^a^1 USD was 22 Ethiopian birr, others@ like Seventh Day Adventist, Jehovah Witness

Income under extreme poverty < 1.25USD per day

Under poverty1.25-2USD per day

Above poverty line >2USD per day

### Obstetric characteristics of pregnant women

One hundred forty four 144(35.8%) were gravida II (had 2nd pregnancy), and 149(37.1%) were para I (the 1st birth including still birth). More than half, 228 (56.7%) of the pregnancy was unplanned. Women in the third trimester of pregnancy who took IFA ≥4 times per week for > 90 days were 154 (38.3%) (95%CI: 33.1, 42.5) (Table 2). Pregnant women who watched medical advice on TV atleast once per two week 236(58.5%) and 233(55.5%) never read medical magazine (Table 1).

**Table 2:** Obstetrical characteristics of pregnant women in Hawassa city, South Ethiopia, 2015

Variable		Number (*N* = 402)	Percent (%)
Number of pregnancy	1	142	35.3
	2	144	35.8
	3–4	99	24.6
	> 4	17	4.2
Number of live birth	Nulipara	114	28.4
	Para I	149	37.1
	Para I-III	124	30.8
	Para IV and above	15	3.7
Number of ANC contact	One times	43	10.7
	Two times	90	22.4
	Three times	129	32.1
	Four times	101	25.1
	Five and above times	39	9.7
Is pregnancy planned?	yes	174	43.3
	no	228	56.7
How many tablets took per day the last ≥90 days	Iron folic acid was taken ≥4 time per week (51 from 90 pill)	154	38.3
	< 4 times per week	248	61.7

### Pregnant women reason of taking IFA

**Fig. 1:**
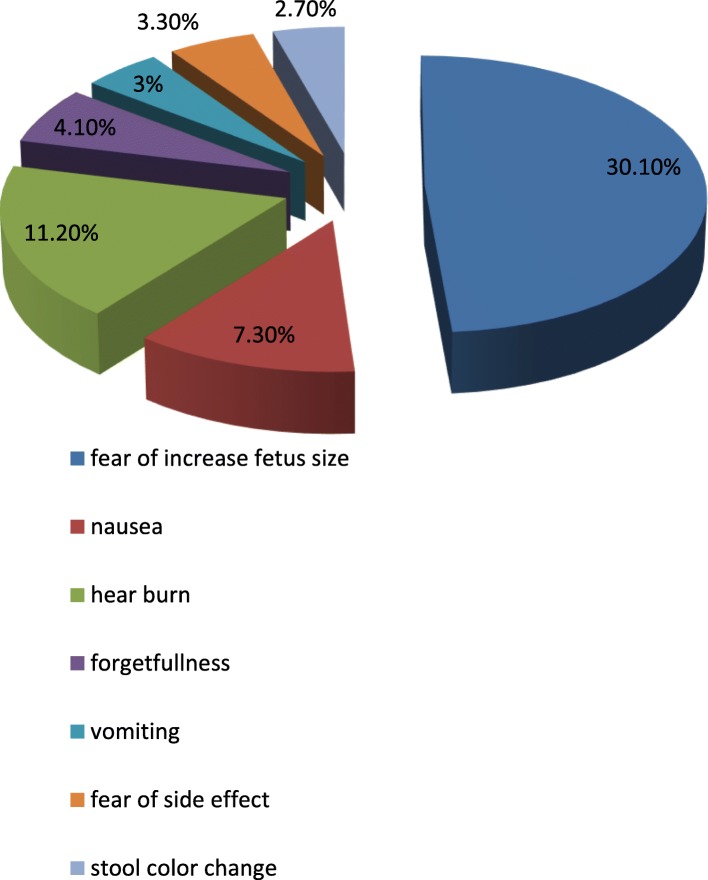
Pregnant women’s the reason of non-compliance of iron folic acid taking in Hawassa city, South Ethiopia, 2015

Among 402 IFA supplement study participant reported that 345(85.8%) took for the prevention anemia (Table 3). Near to one-third of the study participants, 121(30.1%) were feared to fetal size increment was the main mentioned reason of non-compliance with IFA (Fig. 1).

**Table 3:** Pregnant women reason IFA taking in Hawassa city, South Ethiopia, 2015

Variable	Number (*N* = 402)		Percent (%)
Develop complications during the previous pregnancy	Yes	110	27.4
	No	292	72.6
Women who know how to take IFA	Yes	213	53
	No	189	47
Taking IFA for prevention of anemia	Yes	345	85.8
	No	57	14.2
Had anemia during the previous pregnancy	Yes	203	50.5
	No	199	49.5
Discussion the importance of IFA with your friends	Yes	338	84.1
	No	64	15.9
Women who know I the importance of IFA	Yes	199	49.5
	No	203	50.5
Taking IFA for prevention of NTD	Yes	211	52.5
	No	248	61.7

### Factors associated compliance with IFA among pregnant women

Women who know the importance of iron folic acid had 6 time higher odds of compliance with iron folic acid than counterpart (AOR = 6.1, 95% CI: 3.53, 10.24). Pregnant women who develop complication during the previous pregnancy had 0.34 times lower odds of compliance with counterpart (AOR = 0.34, 95% CI: 0.16–0.76), experiencing iron folic acid related side effects during the previous pregnancy had 8.5 times higher odds to decrease compliance with IFA than those who did not experience iron folic acid related side effects (AOR = 8.5, 95% CI: 4.65.-15.35) (Table 4).

**Table 4:** Factors associated compliance with IFA among pregnant women in Hawassa city, South, Ethiopia, 2015(N = 402)

Variable	Compliance (*N* = 154)	Non-compliance (*N* = 248)	COR 95% CI	AOR 95% CI
Discussion the importance of IFA with your friends				
Yes	123	215	1.642 (0.959,2.812)	1.55 (0.74,3.23)
No	31	33	1	
Fearing to take IFA for fetal size increment				
Yes	30	73	0.58 (0.358.0.94) *	1.29 (0.58,2.87)
No	124	175	1	
Develop complications during the previous pregnancy				
Yes	25	85	0.37 (0.225,0.614)*	0.34 (0.16,0.76) *
No	129	163	1	
Is pregnancy planned				
Yes	53	121	0.551 (0.364,0.834)*	1.13 (0.67,1.91)
No	101	127	1	
Experiencing IFA related side effects during the previous pregnancy				
Yes	122	84	7.44 (4.65,11.91) *	8.5 (4.65,15.35)*
No	32	164	1	
Taking IFA for prevention of anemia				
Yes	32	25	2.34 (1.33,4.13) *	1.31 (0.56,2.91)
No	122	223	1	
Had anemia at the previous pregnancy				
Yes	87	112		
No	67	136	1.58 (1.05,2.36) *	0.68 (0.15,3.19)
Women who know the importance of IFA				
Yes	120	83	7.02 (4.42,11.15) *	6.1 (3.53,10.24) *
No	34	165	1	
Women who know how to take IFA				
Yes	71	142	0.64 (0.43,0.96) *	0.47 (0.28,82)
No	83	106	1	

*= > *P* < 0.05

## Discussion

Compliance with IFA is a key strategy of prevention and control of iron deficiency anemia and neural tube defect for pregnant women and unborn child. Nevertheless, it is low in developing countries, including Ethiopia. This study determined the prevalence of compliance with IFA among pregnant women through pill count by direct observation of the pill. It is cost effective and applicable in low and middle income countries to assess compliance. This study showed that compliance with IFA through pills count at third trimester of pregnancy was reported 38.3%.This finding is higher than the study done in Amhara region and compliance with IFA supplementation self-reporting was reported 20.4% [20]. A possible explanation for this higher compliance is might be due to the above study takes place within 12 month after the women gave birth and it is self-reporting of IFA intake while in this study during the pregnancy and pill count by observation. Another possible explanation could be due to study setting could contribute to the findings difference. Thus, this study participant’s access to IFA benefits related health information could be better than the previous study.

This finding is in agreement with the study done in Mangalore, India was 37.1% [21]. (The possible explanation might be happen as acknowledge of the mother related to the use of IFA during pregnancy which enhanced to take IFA properly.

Even though, this finding is inconsistent with the study done in Mangalore India, Mozambique,Vietnam and Madhya Pradesh India 64.7,79,85 and 89% respectively [21–24]. This difference could be explained by sociocultural differences of study groups, study setting and the way of assessing compliance with IFA in different ways. Another possible explanation could be in Ethiopia at public health institution during ANC universally uncoated IFA pill is freely given for pregnant women. This study is in line with the study done in Philippines and Egypt 40.3 and 41.1% respectively through pill count [25, 26]. The possible explanation might be due to the way of defining compliance with IFA through pill count. But lower than the study done in Senegal 69% through pill count [27].

A possible explanation for this lower compliance with IFA might be due to socioeconomic differences of the study groups, time, study setting and population and the previous study assessed compliance with IFA at 20 wk., but this study assessed compliance at third trimester of pregnancy.

This finding is coincided to the study done in Indonesia 36% through stool examination [28]. In this study, the reason of non-compliance is experiencing of side effect, forgetfulness, nausea, heartburn and fear of fetal size increment 3.3, 4.1, 7.3 and 11.2% and 30.1% respectively. It is in line with different studies done [20–25, 27, 29]. A possible explanation might be due to poor counseling of the importance of IFA before prescribing it.

In this study pregnant women who develop complication during the previous pregnancy had 0.34 times lower odds of compliance with counterpart (AOR = 0.34, 95% CI: 0.16–0.76).This finding is coincided to (AOR = 1.52, 95% CI: 1.31–1.75) [30].

In this study experiencing iron folic acid related side effects during the previous pregnancy had 8.5 times higher odds to decrease compliance with IFA than those who did not experience iron folic acid related side effects (AOR = 8.5, 95% CI: 4.65.-15.35), this finding is in line with the study done in Egypt [26, 31]. In this study Women who know the importance of iron folic acid had 6 time of higher odds of compliance with iron folic acid than counterpart (AOR = 6.1, 95% CI: 3.53, 10.24).This finding is in inconsistent to WHO report AOR = 0.05, 95% CI: 0.04,0.07) [29]. This might be happened due to study setting, time, population and the way of addressing compliance with IFA.

The possible strength of this study is used pill count by direct observation. To assess compliance with IFA through pill count by direct observation is cheap and cost effective compared electronics pill count and stool examination. It is applicable in low and middle income countries. The possible limitations of this study did not include that gold standard methods of measuring number of IFA uptake electronics pill count method and stool examination or biochemical methods to measure level of compliance with IFA. Compliance with IFA is one of the strategies to prevent and control iron deficiency anemia and neural tube defect for pregnant women and unborn child [32].

## Conclusion and recommendation

In this study demonstrated that compliance with IFA among pregnant women through pill count is low. Women who know the importance of iron folic acid, women who develop complications during previous pregnancy, experiencing iron folic acid related side effects during the previous pregnancy were independent predictor of compliance with iron folic acid. Health care providers shall strongly counsel the importance and side effect of iron folic acid before prescribing. The responsible bodies avail suitable iron folic acid pill and less side effect brands. Health care providers and health extension workers shall be monitored iron folic acid by pill (tablet) count during their home to home visits.

## Abbreviations

ANC: Antenatal Care
AOR: Adjusted odds ratio
CI: Confidence Interval
EDHS: Ethiopian Demographic and Health Survey
ID: Iron deficiency
IDA: Iron deficiency anemia
NTD: Neural Tube Defect
SD: Standard deviation
SNNPRS: Southern Nations, Nationalities and People Regional State
WHO: World health organization
